# A model of crop diversification under labor shocks

**DOI:** 10.1371/journal.pone.0229774

**Published:** 2020-03-03

**Authors:** Allegra A. Beal Cohen, Jasmeet Judge, Rachata Muneepeerakul, Anand Rangarajan, Zhengfei Guan

**Affiliations:** 1 Department of Agricultural and Biological Engineering, University of Florida, Gainesville, Florida, United States of America; 2 Center for Remote Sensing, Agricultural and Biological Engineering, University of Florida, Gainesville, Florida, United States of America; 3 Computer & Information Science & Engineering, University of Florida, Gainesville, Florida, United States of America; 4 Food and Resource Economics, University of Florida, Gainesville, Florida, United States of America; University of Palermo, ITALY

## Abstract

As demands on agriculture increase, food producers will need to employ management strategies that not only increase yields but reduce environmental impacts. Modeling is a powerful tool for informing decision-making about current and future practices. We present a model to evaluate the effects of crop diversification on the robustness of simulated farms under labor shocks. We use an example inspired by the Florida production system of high-value, labor-intensive fruits. We find that crop diversification to high-value crops is a robust strategy when labor shocks are mild, and that crop diversification becomes less valuable as more simulated farms practice it. Based on our results, we suggest that crop diversification is a useful management strategy under specific conditions, but that policies designed to encourage crop diversification must consider broad effects as well as farm-level benefits.

## Introduction

The estimated increase in global food demand over the next thirty years ranges between 70% and 100% of current production. To match increasing demand, agricultural management strategies will need to adapt to produce more food with less environmental impact [1]. Better management practices can positively impact climate change and food security. For example, soil management, such as no-till farming and cover crops, could offset 5 to 15% of current global emissions through carbon sequestration [2]. However, it is not always feasible to evaluate management practices through field trials, especially for multiple locations and crops, and it is often difficult to answer questions about the future. Modeling is a powerful tool for formalizing complex problems and informing decisions about current and future practices. A long history of agricultural systems modeling shows that modeling can be used both to increase understanding for scientists and support policy-making about how food is grown [3].

Agricultural models have often been used to assess the potential of agricultural policies and management strategies such as integrated pest management and cover cropping [4, 5]. However, one strategy with little presence in models is crop diversification, despite many studies showing its benefits. Crop diversification can enhance both the visual and environmental properties of landscapes while reducing economic risks to farmers, and has been a part of attempts to reduce the environmental impact of agriculture [6–9]. Agricultural biodiversity has been found to increase the resilience of agricultural landscapes in a review of 172 case studies and project reports from around the globe [10]. Di Falco and Chavas have shown that crop biodiversity reduces the cost of risk in Ethiopia and increases profitability in Bulgaria [11, 12]. Crop diversification has been suggested as a strategy to mitigate the effects of climate change and weather patterns such as the El Niño-Southern Oscillation, and to improve the economic resilience of an agricultural landscape [13–15]. Seo (2010) found that for climate predictions in 2060, African farms are predicted to diversify between crops and livestock, and profit better than those that remain specialized; however, theirs was a behavioral model, not an agricultural model [16]. Gimona and Polhill (2011) evaluated the effects of incentive programs on agricultural landscape biodiversity with a coupled agent-based model of species and farms; they found that the success of incentive schemes is dependent on the species being protected and the characteristics of the region [17]. These papers and a growing literature of land-use and agricultural policy models show the value of modeling agricultural practices, but a gap remains for models of crop diversification under disturbances [18]. In this paper, we evaluate the use of crop diversification as a management strategy in order to close this gap.

Models which evaluate management strategies like crop diversification often do so in response to changing circumstances, such as climate change and price volatility. Few models address labor supply directly as a factor in farmer decision-making, despite the importance of labor in the production of many fruit and vegetable crops. Labor makes up a significant portion of U.S. fruit and vegetable farms’ expenses, and this portion has steadily increased; at the same time, mechanization is still not an economical option for many farmers, as tasks involving high-value fruits and vegetables are technically difficult [19, 20]. Thus, modeling the effects of disruptions in labor supply due to wage increases or immigration policy changes is important, given the large costs of labor to farmers. Messina, Letson and Jones (2007) modeled labor as a limiting factor in farm decision-making, allowing a labor quota for each farm based on availability estimated using historical shipments. They found that the value of ENSO forecasts increased when more labor was available at planting time, and the value was positive in all labor scenarios [21]. White, Labarta and Leguía (2005) studied the effects of seasonal labor shortages on technology adoption among resource-poor farmers [22]. They found that when integrating new technology into resource-poor areas, researchers and policymakers must account for the new demands on already scarce labor; otherwise, only wealthier farmers might adopt the technology. Because labor availability is a limiting factor in many agricultural systems, especially those which require manual harvesting, labor should be included in models which evaluate the usefulness of management strategies. We address this gap by evaluating crop diversification with respect to labor shortages.

While many agricultural models are at the field level, some are used to ask questions at the farm scale and higher, especially when the modeler is interested in policy. Gimona and Polhill (2011) found that rewarding simulated farms for the behavior of their neighbors drastically increased the success of species-protection incentive schemes, but that the balance between the financial cost of the scheme and its success was delicate [17]. Messina, Letson and Jones (2006) examined the usefulness of El Niño-Southern Oscillation (ENSO) forecasts at the farm and regional scale for the Florida fresh tomato industry. Using farmer interviews and the crop growth model CROPGRO-Tomato, they modeled farmer decision-making at the farm scale and estimated its effects at the regional scale through market changes and farmer cooperation. They found that while forecasts could benefit individual farms economically, widespread adoption of forecasts countered those benefits [21]. Thus, when building a model of decision-making at the farm scale, it is necessary to look at the cumulative effects of individual decisions. However, the effects of crop diversification on farms under labor shortages have not yet been addressed at multiple scales.

In this paper, we address the aforementioned gaps in models of agricultural systems using our farm- and regional-scale model. The effects of crop diversification during labor shortages has not yet been studied at multiple scales; however, expanding our understanding of when crop diversification is useful can provide baseline guidance for policy- and decision-making in real world scenarios. We address the robustness of crop diversification as a strategy under labor shocks. While much work has been done on the resilience of agricultural systems [23–26], little work focuses on robustness. Robustness is a useful concept for guiding decision-making about management strategies, because it refers to how a system responds to perturbations or uncertainty. While engineering resilience is often defined as the rate at which a system returns to equilibrium after a disturbance [27], Carlson and Doyle (2002) define robustness in engineering systems as the persistence of desired system characteristics despite internal fluctuations or external disturbances [28]. Homayounfar *et al*. (2018) summarize robustness as the sensitivity of a system’s performance to disturbances, which is the definition we use in this study [29].

We present a multi-scale stylized model to explore when crop diversification is a robust farming strategy during labor disturbances. The goal of the model is to evaluate robustness at the farm and regional scales under a variety of labor shortages, in order to understand how crop diversification affects robustness at multiple scales. We simulate farming units under different types of labor shocks and determine how individual units’ strategies affect regional robustness, using a case study inspired by the Florida production system. To conclude, we discuss our results as well as the limitations and future implementations of our model.

## The model

**Fig 1:**
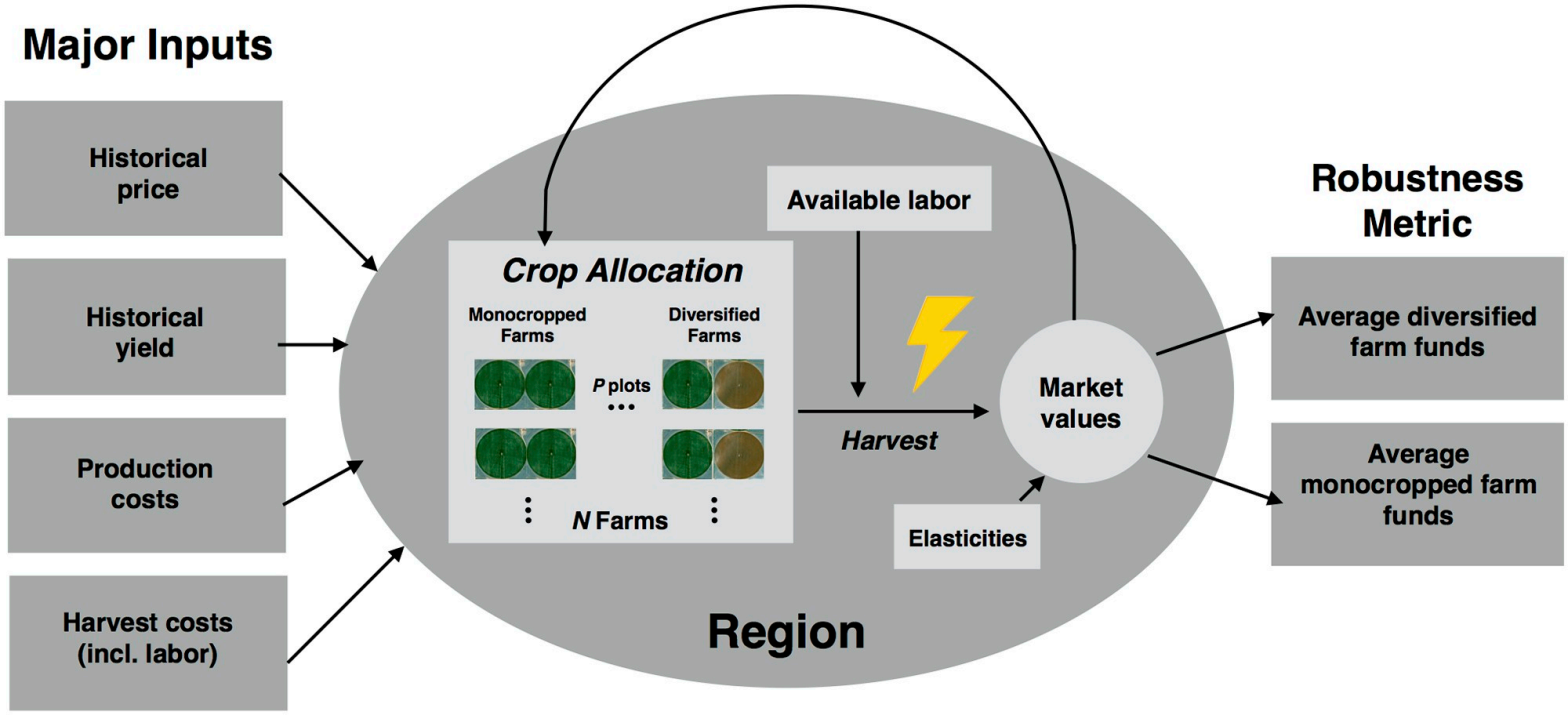
Flow chart of the inputs, outputs and interaction factors of the model. The lightning bolt indicates only a fraction of baseline labor is available (a “shock”). The events within the gray oval, such as crop allocation, harvest, and the change in market prices, define a season.

Fig 1 shows the components of our model. It describes a region consisting of *N* farming units, each of which has *P* plots of S_1_, …, S_P_ acres. Each plot must be planted in its entirety with a single crop. In addition to the number of plots, the farming units in the region are characterized by the yields of each plot (kg/acre); the production and harvest costs per acre for each crop available to the farmer; the number of laborers required to harvest an acre of each crop; initial funds; and the farm’s planting strategy (“monocropped” or “diversified”). Production costs include transplants, herbicides, fertilizer, equipment, and other aspects of production; harvest cost includes only the cost of labor used in harvest. In each time step, farming units harvest crops based on their market prices and the amount of available labor; that is, partial harvests are possible. The total yields harvested by all farming units in the region are used to calculate the market prices of the crops in the next season. This allows exploration of the effects of individual farming units’ strategies on regional market price, which in turn is an important economic factor in the robustness of agricultural systems.

The model has three important variables at the regional scale: The market prices for each crop; elasticities of demand for each market price; and the labor available for each season (“available labor” in Fig 1). Seasonal labor shortages are called “shocks”, and can be of varying intensity (*i*) and duration (*d*). The model is used to run simulations of *τ* seasons, with one season lasting from planting to the change in market prices when the crops have been harvested, as shown in the gray oval in Fig 1.

### Interaction among farming units within the region

Farming units interact by proxy through changing market prices. For example, a large number of farming units growing Crop 1 might be expected to lower the market price of Crop 1 and therefore bolster the relative profits of farming units growing Crop 2. We approximated an empirical market price equation for our case implementation using the mean market price and yield of the case crops from 2000 to 2009 [30, 31]. At the end of each season, market prices (V) are calculated with the elasticity of demand and the simulated yield harvested for each crop, as shown in Eq (1):

$$\begin{array}{c} V_{t}=a\cdot \left(Y_{t}^{\epsilon }\right) \end{array}$$ (1)

where *V*_*t*_ is the market price of a crop at the end of season *t*; *a* is a constant derived from historical yield and market price data; *ϵ* is the elasticity of the crop; and *Y*_*t*_ is the total amount of crop harvested (kg) by all farming units in season *t*. The market prices calculated with Eq (1) are used to determine farming units’ actual profits in season *t*, as well as acting as expected prices during crop allocation for season *t + 1*. Because it aggregates the yields of all farming units, Eq (1) is the primary mechanism by which an individual farming unit is influenced by the decisions of other units.

### Decision-making at the farming unit scale

#### Planting decisions

At the beginning of a season, farming units assess their planting options using a constrained integer programming optimization algorithm similar to that developed by Radulescu [32]. Using the market prices calculated with Eq (1) for season *t* as the expected market prices for season *t+1*, farming units maximize their expected profits by allocating crops to plots. Let *x*_*ij*_ be the binary variable representing whether a farming unit plants crop *i* on plot *j*. Then the farming unit aims to maximize its profit through the allocation **x** of *m* crops to *n* plots:

$$\begin{array}{c} \begin{array}{cc} & \text{max}(\Psi (x))\\ & \text{subject}\;\text{to}\\ & \sum_{i=1}^{m}x_{ij}\leq 1,\text{for}\;\text{every}\;\text{j}\in \left\{1,2,\ldots ,n\right\}\\ & x_{ij}\in \left\{0,1\right\},i\in \left\{1,2,\ldots ,m\right\},j\in \left\{1,2,\ldots ,n\right\} \end{array} \end{array}$$

where each plot can have no more than one crop, and profit is calculated as:

$$\begin{array}{c} \Psi \left(x\right)=\sum_{i=1}^{m}\sum_{j=1}^{n}\left(V_{i}Y_{ij}h_{i}L_{ij}x_{ij}-C_{L}L_{ij}x_{ij}-a_{ij}x_{ij}\right) \end{array}$$ (2)

*V*_*i*_ is the market price of crop *i*, *Y*_*ij*_ is the yield for crop *i* on plot *j*, and *h*_*i*_ is the harvest efficiency of crop *i*, or the number of acres of crop *i* that can be harvested with one unit of labor. *L*_*ij*_ is the amount of labor that will be required to harvest crop *i* on plot *j*, *C*_*L*_ is the cost of labor, and *a*_*ij*_ represents the production costs other than labor. To simplify our case implementation, we restricted farming units to one crop (“monocropped”) or two (“diversified”) and required strategies to remain constant throughout a simulation, e.g. a farming unit that plants a single crop in one time step cannot diversify in the next.

#### Harvesting decisions

At harvest time, farming units harvest their crops based upon the crops’ labor requirements, the available labor for that season, and the expected market price. Farming units would like to maximize profit as in Eq (2), but instead of maximizing through crop allocation, they now optimize the allocation of labor (*L*_*ij*_) to crops and plots. Monocropped farming units only harvest as much of their single crop as the expected market price dictates to be profitable and as labor allows. Consider an example with two crops (Crop 1 and Crop 2), where farming units each have two plots, designated 1 and 2. A monocropped farming unit might harvest only part of its crop during a labor shortage. A diversified farming unit can maximize profits by partitioning its labor between its two crops based on the expected market price. Eq (3) shows the expected profit of a diversified farming unit (Ψ_*D*_) which plants Crop 1 on its first plot and Crop 2 on its second plot, and Eq (4) shows the expected profit of a monocropped farming unit (Ψ_*M*_) that plants only Crop 1 on both plots:

$$\Psi_{D}=V_{1}Y_{1,1}h_{1}L_{1,1}+V_{2}Y_{2,2}h_{2}L_{2,2}-C_{L}\left(L_{1,1}+L_{2,2}\right)-\left(a_{1,1}+a_{2,2}\right)\;$$ (3)

$$\Psi_{M}=V_{1}\left(Y_{1,1}L_{1,1}+Y_{1,2}L_{1,2}\right)h_{1}-C_{L}\left(L_{1,1}+L_{1,2}\right)-\left(a_{1,1}+a_{1,2}\right)$$ (4)

where the total amount of labor allocated by a farming unit must be less than or equal to the amount of labor available to each farming unit at harvest time. For simplicity, in this study it is assumed that each farming unit has access to the same amount of labor, given that no farm in reality typically pays more than minimum wage. After the market prices of crops are updated based upon collective yields in the region (Eq (1)), each farming unit updates its funds for next season with the profit (Ψ) it made during the season.

### Robustness quantification

The relative robustness of the monocropping and diversification strategies are quantified through the average funds of the farming units which employ each strategy. Farms with greater average funds are more robust—i.e., less sensitive—to the financial consequences of labor shocks. We define a labor shock as the fraction *i* of total labor, $\sum_{f=1}^{N}L_{A_{f}}$, available to the region for some number of seasons *d*. Fig 1 shows the major inputs to the model and the robustness metric.

## Methodology

### Case implementation

Although our model is flexible enough to simulate different types of farms, our case implementation focuses on two labor-intensive crops with data from the Florida strawberry and tomato production systems. We examine the robustness of diversified farming units relative to monocropped farming units, where “diversification” means growing both strawberries and tomatoes.

Florida is an important contributor to the national supply of strawberries, a crop which is both high-value and labor-intensive. Florida is the second largest producer of strawberries in the U.S., with a farm gate value of $300 million [33]. The primary problem facing Florida strawberry farmers is lack of labor, as the crop must be hand-picked. Labor costs for strawberry farmers rose 32% between 2008 and 2013, with 21% of that increase due to factors other than yield [33]. Most of a farmer’s labor supply is composed of migrant agricultural workers, primarily from neighboring Mexico, working for minimum wage. As Mexico’s economy and job opportunities improve, the U.S.’s labor supply coming from Mexico may decrease. Increasingly strict immigration policy is also a major influence [20]. Tomatoes are widely grown in Florida as well. In 2015, Florida tomato farms produced $453 million or 36% of the U.S. value for fresh market tomatoes [34]. However, tomatoes require less labor than strawberries to harvest. The vulnerability of Florida’s strawberry system to labor shortages and the widespread planting of both crops made them an appropriate example for our model.

In this study, we ask under what type of labor shocks is crop diversification a more robust strategy for farming units than strawberry monocropping. While the expected answer might be that diversification is always more robust for any given farming unit, Messina, Letson and Jones (2006) showed that when regional interactions are accounted for, assumptions at the individual scale are not always correct. We parameterized the model with data from the Florida strawberry and tomato systems; Table 1 shows the model inputs specific to Florida tomato and strawberry producers and their data sources. These sources are a combination of government statistics and academic papers. We selected the academic papers based on relevance, the specificity of many of their findings to the Florida production system, and their high level of detail. In particular, Guan, Wu, and Whidden (2017) and Guan, Wu, and Sargent (2017) provide detailed and recent statistics on labor costs for Florida strawberry and tomato production.

**Table 1:** Inputs to the model.

Input	Florida case values	Source
Initial farming unit funds	$1,871,571	[35]
*Tomatoes*		
market prices	-	Available for download at [31]
Elasticity	-0.58	[36]
Production cost not incl. harvest	10,078 (USD/acre)	[37]
Labor cost	2408 (USD/acre)	[37, 38]
Labor requirement	29 (people/100 acre)	[38, 39]
*Strawberries*		
market prices	-	Available for download at [30]
Elasticity	-0.66	[40]
Production cost not incl. harvest	12,305 (USD/acre)	[33]
Labor cost	7788 (USD/acre)	[33]
Labor requirement	95 (people/100 acre)	[39]

Each simulated farming unit has two 100-acre plots on which to plant; every farming unit has the same amount of acreage. The strategies are to plant strawberries on both plots or tomatoes on both plots (“monocropped”), or one on each (“diversified”). The ratio of plot sizes dedicated to strawberries and tomatoes affects model output linearly. We chose equal-sized plots to maximize the difference between a diversified farming unit and a monocropped farming unit of either type. The yields of these plots at the end of each season are the same for each farming unit. Similarly, each farming unit receives an equal amount of labor with which to harvest, although the total amount available to the region varies based on the simulation’s labor shock.

### Labor shocks

14 sets of simulations were run to examine the robustness of crop diversification under shocks of different intensities and durations, as well as with different numbers of diversified farming units in the region. The region in each set of simulations comprised 30 farming units with a different number of diversified units ranging between 2 and 28 in multiples of 2. The monocropped farming units were split evenly between strawberry and tomato planters to balance the monocropped farming units’ effects on the market prices of each crop. For example, one set of simulations was run with 2 diversified units, 14 strawberry units and 14 tomato units; the next was run with 4 diversified units, 13 strawberry units and 13 tomato units; and so on.

Each set of simulations consisted of 100 combinations of intensities and durations of labor shocks. All simulations were run for *τ* seasons, with *τ* being the duration (*d*) of the labor shock plus one season, and *N* = 30 farming units. Labor shocks always began in the first season of the simulation. The baseline labor ($\sum_{f=1}^{N}L_{A_{f}}$) available to the region was calculated by taking the harvest labor requirement per acre of strawberries (the most labor-intensive crop) and multiplying it by the number of farming units and acres per farming unit, to ensure enough labor for all units. Shocks were calculated by multiplying baseline available labor by *i* = 0, 0.1, …, 1.0. More “extreme” shocks are indicated by a lower *i*, e.g. *i* = 0.1 means that the region has 10% of its usual labor available. Shocks were simulated for durations of *d* = 1 to *d* = 10 seasons. After running all simulations, we analyzed the data for points at which diversified farming units’ funds became larger than strawberry or tomato farming units’ funds. The purpose of this was to define labor scenarios in which diversified farming units held a robustness advantage against monocropped units.

## Results

Our 14 simulation sets show that diversification increased farming units’ robustness relative to monocropped competitors when shocks were mild (*i* > 0.2 for strawberry planters and *i* > 0.5 for tomato planters) and when the number of diversified farming units was low. In some cases, diversified farming units performed better than both strawberry and tomato monocropping units, and in other cases, they performed better than only strawberry monocropping units. In this discussion we focus on the intensity of shocks and the number of diversified farming units in the region. The duration of shocks affected the magnitude of farm funds, as the longer a simulation ran the more funds a farming unit accumulated, but did not change which units were more robust. This was because no farming units exited production during any simulation under the parameterization shown in Table 1.

### Intensity of shock

**Fig 2:**
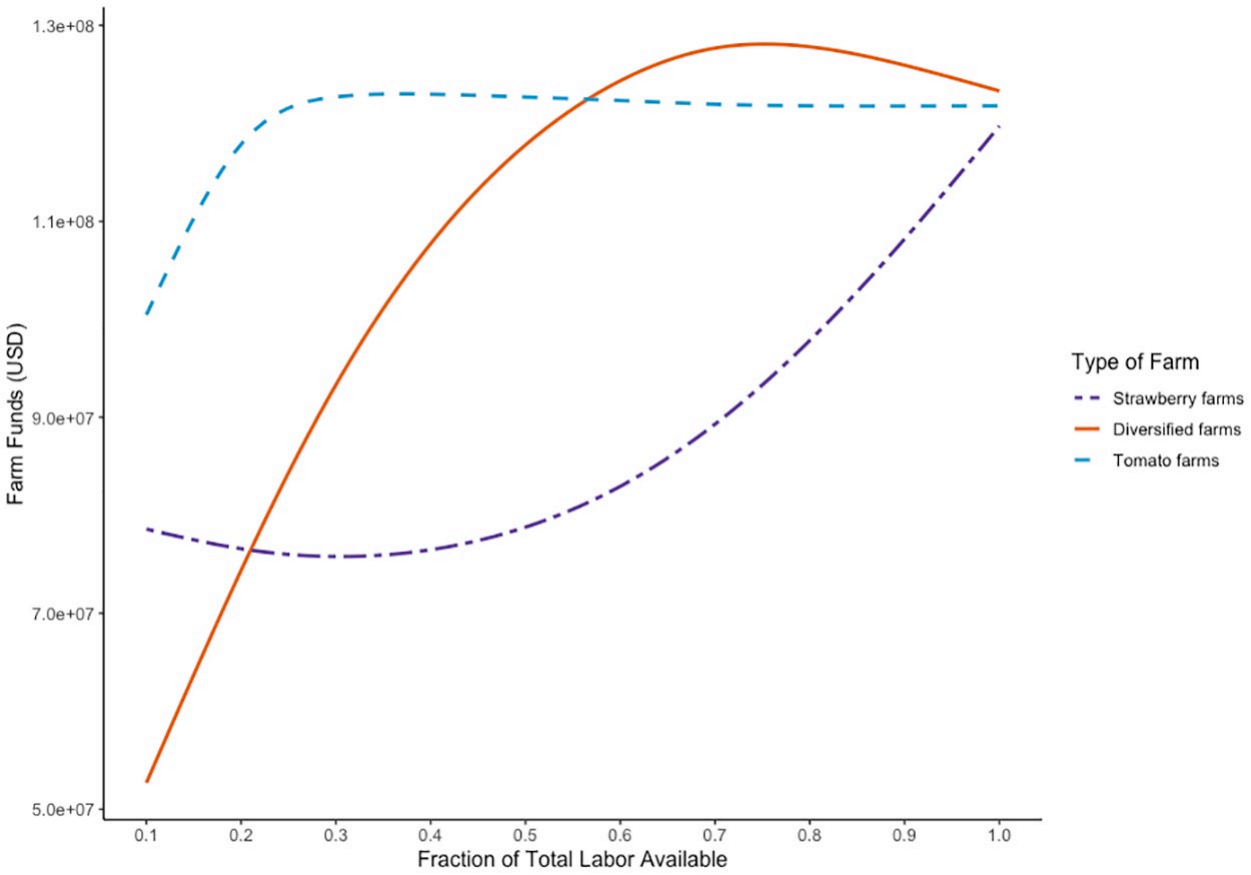
Farm funds under different shock intensities. The x-axis shows shock intensities. The y-axis shows the average funds for strawberry, diversified, and tomato farming units indicated by the line type and color. Lines are smoothed to reveal trends.

The lines in Fig 2 summarize simulations run with the shock intensities indicated on the x-axis. The y-axis shows the mean funds for farming units of the strawberry, diversified, or tomato-growing type, indicated by the color and type of line. Fig 2 shows that under shocks with intensities of $i<0.5\cdot \sum_{f=1}^{N}L_{A_{f}}$, tomato farming units consistently did better than diversified units. Tomato farming units maintained consistent funds under shocks with more than 20% of labor available because they were able to harvest all their crop; this was because labor shocks were calculated based on the requirements of the higher-labor crop, strawberries. Under milder shocks with intensities of $0.6>\cdot \sum_{f=1}^{N}L_{A_{f}}$, diversified farming units began to have an advantage over tomato units.

Tomato farming units were more robust under extreme (*i* < 0.5) shocks than diversified units because the latter did not harvest enough strawberries to make up the difference in profits. While strawberries had a higher market price, they required more labor to harvest. Only under milder shocks did diversified farming units have enough labor to optimize between their crops. Without enough labor, diversified farming units acted as smaller monocropped units, harvesting tomato plots that were half as large as those harvested by monocropped tomato farming units. With enough labor, under shocks with *i* > 0.5, diversified units harvested enough strawberries to take advantage of that crop’s greater market price. Additionally, when 0.2 < *i* < 0.6, diversified farming units were still more robust than strawberry units; they harvested enough tomatoes to provide an advantage over strawberry farming units, though not enough strawberries to surpass tomato farming units.

### Number of diversified farming units

**Fig 3:**
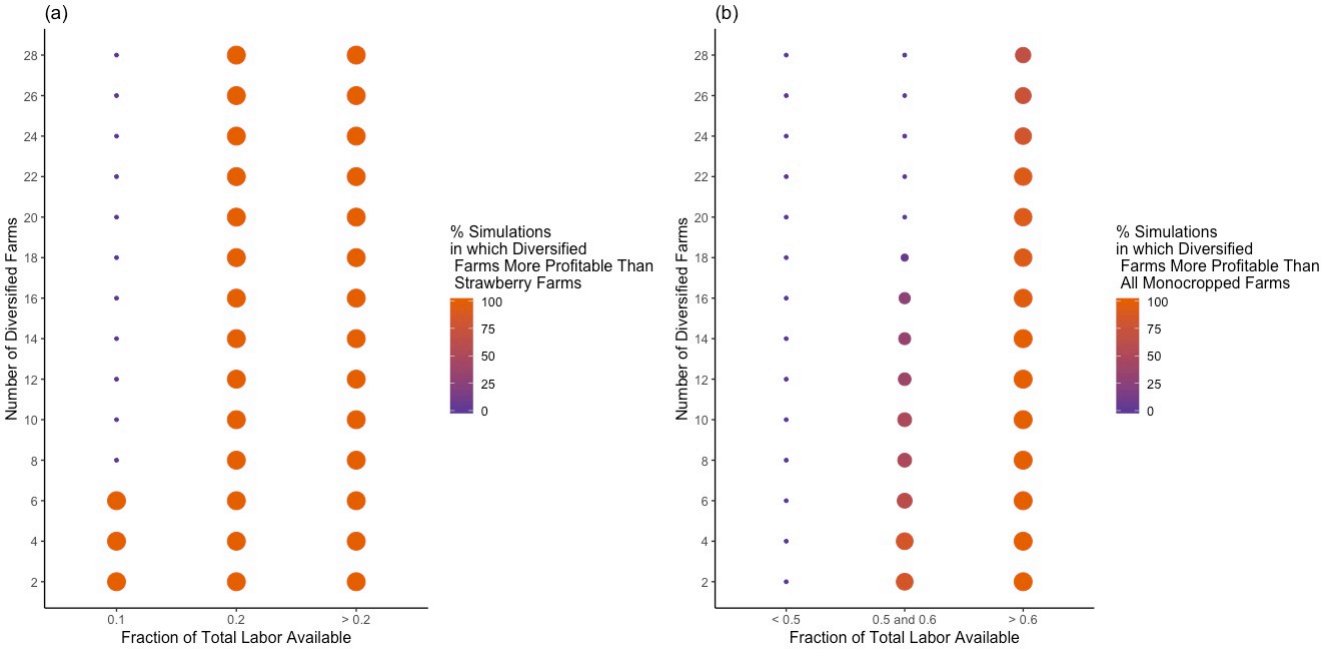
Diversification advantage by number of diversified farms and intensity of shock. On the y-axes are the number of diversified farming units in each simulation. On the x-axes, the intensity of shocks in each simulation are broken up into relevant segments to simplify the figure. Points represent simulations with the corresponding number of diversified farming units and intensity of shock. The color and size of points represent the percentage of simulations in which diversified farming units do better than (a) strawberry units and (b) strawberry and tomato farming units.

The advantage of diversification also depended on the number of diversified farming units in the region. Fig 3 shows the parameters under which diversified farming units were more robust than (a) only strawberry farming units and (b) all monocropped farming units. The color and size of the points correspond with the percentage of simulations in which diversified farming units’ profits were larger than (a) strawberry units’ and (b) all monocropped units’ profits. The percentage of simulations in which diversified farming units were more robust than all other units increased as the number of diversified units decreased, a finding that corresponds with those of Messina, Letson and Jones [21].

Only simulations with a very small number of diversified farming units showed diversification as a more robust strategy than strawberry monocropping under *i* = 0.1 shocks, as can be seen in Fig 3a. As shocks became milder in intensity, more diversified farming units could adopt the strategy profitably. Fig 3b also shows that diversification was more effective when there were fewer diversified farming units. The number of diversified farming units did not significantly affect mean regional wealth under any type of shock. Thus, diversification was a valuable strategy to individual farming units, but did not contribute to regional robustness under the conditions of our model.

### Discussion

Our model integrates the farm and regional scales, which allows us to make inferences about the robustness of both individual farming units and about the region as a whole. At the farming unit scale, we found that farming units which use crop diversification are more robust when labor conditions are appropriate. In the case of farming units that plant lower-value crops, diversification to a higher-value crop can increase robustness under mild labor shocks. For farming units that plant high-value crops, diversifying to crops that require less labor can benefit units during shortages even if those crops are of lower value. These findings address a significant gap in agricultural models that include both crop diversification and labor supply.

Few agricultural models evaluate management practices on multiple scales, but our model addresses this gap and shows that the interaction of farming units at the regional scale is important. The advantages of crop diversification at the farming unit scale are not reflected perfectly in results at the regional scale. We found that the value of diversification as a robust response to labor shortages decreased when too many farming units adopted diversification. That is, an advantage for every participant in the market was no advantage at all. Our findings support those of Messina, Letson and Jones [21]. These results demonstrate that this effect can occur for different kinds of individual-level advantages, from information to management practices.

Agricultural models are useful for assessing management practices; however, few models evaluate crop diversification on multiple scales, despite many studies indicating the benefits of agricultural biodiversity [4–6, 8, 11]. Moreover, labor availability is often left out of agricultural models, although it is an important factor for farmers [21, 22]. Our model addresses these gaps by including both crop diversification and labor availability. The results of our study confirm that crop diversification can be a robust way to distribute risk for individual farming units under labor shortages. Nonetheless, even at the farming scale, crop diversification was not always the most robust strategy; the crop with which farms diversify must correspond to the level of labor available. For example, our results did not show an advantage for diversified farming units over tomato farming units during extreme labor shortages, because strawberries required too much labor to harvest despite their higher value. Thus, when considering ways for farms to improve robustness to disturbances, it is important to ensure that the strategies selected are appropriate.

Our study also suggests that when designing policy or extension programs to increase the robustness of a production system, designers ought to be aware that economic advantages can decrease with the number of farms adopting a strategy. We demonstrated that widespread crop diversification did not improve the robustness of diversified farming units as much as when the strategy was rare. However, we did not study the other potential benefits of crop diversification, and we defined robustness in economic terms. For example, if a region of farms adopted crop diversification to reduce pest load, widespread adoption might make the region more robust to pests, similar to the way that clustered incentive schemes increased species persistence in the Gimona and Polhill (2011) model [17]. Future work should consider multiple effects of management practices outside of economic measures.

Our stylized model offers a clear integration of farm- and regional-level dynamics through market price. However, our model’s economic component is simple and straightforward, and some structural realism may be lost. For example, the duration of the labor shock in our simulations did not affect robustness, as no farming units went out of business. Given that real farms do go out of business, we note that our model may not capture the nuances of the Florida production system. In particular, we overlook factors which tend to affect labor supply, such as crop seasonality. Moreover, although the number of diversified farming units affected the robustness of those units, regional robustness was unaffected. This is due to the fact that in our model, an increase in profit for farming units using one strategy corresponded to a decrease in profit for other farms, given the restriction of farms to two crops and two market prices. Thus, while stylized models have the advantage of clarity, ours is also limited in its representation of economic dynamics.

## Conclusion

We used our model to compare the robustness of diversified and monocropped farming units to different types of labor shocks in an example inspired by the Florida production system. Our model addresses gaps in the modeling literature for crop diversification, labor shortages, and the effects of these on the robustness of agricultural systems. We found that diversification was a robust strategy under labor shocks where more than half the usual labor force was available, but that the advantages of diversification decreased with the extremity of the labor shortage. These findings suggest the importance of modeling the interaction between management practices and external factors, as the level of labor availability affected the usefulness of the crop diversification strategy. We also examined the regional interactions between farming units, and showed that the value of diversification decreased rapidly as the number of diversified farms increased. That crop diversification did not offer a linear robustness advantage in simulations was not initially expected, and points to the necessity of modeling at multiple scales. Our findings imply that those recommending crop diversification as a way to increase farms’ robustness ought to consider wider system dynamics.

Although our model has contributed to the evaluation of management practices, its design is somewhat limited. Our stylized model offers a clear picture of system dynamics without the need for quantitative time series data, but it sacrifices some structural fidelity. A more complex implementation of the model may offer different insights and continue to improve the literature on modeling management practices at multiple scales. As discussed, an improved economic component would allow better analysis of total regional robustness, as well as potentially allow the model to be used for predictions.

Our model also made several simplifying assumptions: Farming units were identical other than their cropping strategies; strategies could not change over time; and information about market price was perfect. Removing any of these assumptions could result in interesting implications for policy and extension program design, as well as improve the structural realism of the model. Real farms are rarely identical in size, financial situation, environmental characteristics, or management practices. Allowing these characteristics to vary would show how management strategies might affect the robustness of farming units differently, especially if multiple aspects of strategies were included, such as the usefulness of crop diversification for managing pests. Moreover, changing the quality and quantity of information about strategies as well as the ability of farming units to select their strategies might allow researchers to model the adoption of new practices.

## References

[1] Godfray HCJ, Beddington JR, Crute IR, Haddad L, Lawrence D, Muir JF, et al Food security: The challenge of feeding 9 billion people. Science. 2010;327(5967):812–818. 10.1126/science.1185383 20110467

[2] Lal R. Soil carbon sequestration impacts on global climate change and food security. Science. 2004;304(5677):1623–1627. 10.1126/science.1097396 15192216

[3] Jones JW, Antle JM, Basso B, Boote KJ, Conant RT, Foster I, et al Brief history of agricultural systems modeling. Agricultural Systems. 2017;155:240–254. 10.1016/j.agsy.2016.05.014 28701816 PMC5485640

[4] Laborte AG, Van Ittersum MK, Van den Berg MM. Multi-scale analysis of agricultural development: A modelling approach for Ilocos Norte, Philippines. Agricultural Systems. 2007;94(3):862–873. 10.1016/j.agsy.2006.11.011

[5] Gibbons JM, Sparkes DL, Wilson P, Ramsden SJ. Modelling optimal strategies for decreasing nitrate loss with variation in weather–a farm-level approach. Agricultural Systems. 2005;83(2):113–134. 10.1016/j.agsy.2004.02.010

[6] Cortignani R, Gobattoni F, Pelorosso R, Ripa M. Green Payment and Perceived Rural Landscape Quality: A Cost-Benefit Analysis in Central Italy. Sustainability. 2018;10(8):2910 10.3390/su10082910

[7] Solazzo R, Donati M, Tomasi L, Arfini F. How effective is greening policy in reducing GHG emissions from agriculture? Evidence from Italy. Science of The Total Environment. 2016; 573:1115–1124. 10.1016/j.scitotenv.2016.08.066 27694042

[8] Altieri MA. The ecological role of biodiversity in agroecosystems. Invertebrate Biodiversity as Bioindicators of Sustainable Landscapes. 1999;19–31. 10.1016/B978-0-444-50019-9.50005-4

[9] Lin BB. Agroforestry management as an adaptive strategy against potential microclimate extremes in coffee agriculture. Agricultural and Forest Meteorology. 2007;144(1-2):85–94. 10.1016/j.agrformet.2006.12.009

[10] Mijatović D, Van Oudenhoven F, Eyzaguirre P, Hodgkin T. The role of agricultural biodiversity in strengthening resilience to climate change: towards an analytical framework. International Journal of Agricultural Sustainability. 2013;11(2):95–107. 10.1080/14735903.2012.691221

[11] Di Falco S, Chavas JP. On crop biodiversity, risk exposure, and food security in the highlands of Ethiopia. American Journal of Agricultural Economics. 2009;91(3):599–611. 10.1111/j.1467-8276.2009.01265.x

[12] Di Falco S, Penov I, Aleksiev A, Van Rensburg TM. Agrobiodiversity, farm profits and land fragmentation: Evidence from Bulgaria. Land Use Policy. 2010;27(3):763–771. 10.1016/j.landusepol.2009.10.007

[13] Lin BB. Resilience in agriculture through crop diversification: Adaptive management for environmental change. BioScience. 2011;61(3):183–193. 10.1525/bio.2011.61.3.4

[14] Messina C, Hansen J, Hall A. Land allocation conditioned on El Niño-Southern Oscillation phases in the Pampas of Argentina. Agricultural Systems. 1999;60(3):197–212. 10.1016/S0308-521X(99)00032-3

[15] Abson DJ, Fraser ED, Benton TG. Landscape diversity and the resilience of agricultural returns: a portfolio analysis of land-use patterns and economic returns from lowland agriculture. Agriculture & Food Security. 2013;2(1):2 10.1186/2048-7010-2-2

[16] Seo SN. Is an integrated farm more resilient against climate change? A micro-econometric analysis of portfolio diversification in African agriculture. Food Policy. 2010;35(1):32–40. 10.1016/j.foodpol.2009.06.004

[17] Gimona A, Polhill JG. Exploring robustness of biodiversity policy with a coupled metacommunity and agent-based model Journal of Land Use Science. 2011;6(2-3):175–193. 10.1080/1747423X.2011.558601

[18] Matthews RB, Gilbert NG, Roach A, Polhill JG, Gotts NM. Agent-based land-use models: a review of applications Landscape Ecology. 2007;22(10):1447–1459. 10.1007/s10980-007-9135-1

[19] Calvin L, Martin PL. Labor-intensive US fruit and vegetable industry competes in a global market. 2010.

[20] Guan Z, Wu F, Roka F, Whidden A. Agricultural labor and immigration reform. Choices. 2015;30(4):1–9.

[21] Messina C, Letson D, Jones JW. Tailoring management of tomato production to ENSO phase at different scales. Transactions of the ASABE. 2006;49(6):1993–2003. 10.13031/2013.22280

[22] White DS, Labarta RA, Leguía EJ. Technology adoption by resource-poor farmers: considering the implications of peak-season labor costs. Agricultural Systems. 2005;85(2):183–201. 10.1016/j.agsy.2004.07.018

[23] Lien G, Hardaker JB, Flaten O. Risk and economic sustainability of crop farming systems. Agricultural Systems. 2007;94(2):541–552. 10.1016/j.agsy.2007.01.006

[24] Rodriguez D, Power B, Cox H, Crimp S, Meinke H, et al The intrinsic plasticity of farm businesses and their resilience to change. An Australian example. Field Crops Research. 2011;124(2):157–170. 10.1016/j.fcr.2011.02.012

[25] Rodriguez D, Cox H, Power B, et al A participatory whole farm modelling approach to understand impacts and increase preparedness to climate change in Australia. Agricultural Systems. 2014;126:50–61. 10.1016/j.agsy.2013.04.003

[26] Sabatier R, Joly F, Hubert B. Assessing both ecological and engineering resilience of a steppe agroecosystem using the viability theory. Agricultural Systems. 2017;157:146–156. 10.1016/j.agsy.2017.07.009

[27] Holling Crawford S. Resilience and stability of ecological systems. Annual Review of Ecology and Systematics. 1973;4(1):1–23. 10.1146/annurev.es.04.110173.000245

[28] Carlson JM, Doyle J. Complexity and robustness. Proceedings of the National Academy of Sciences. 2002;99(1):2538–2545. 10.1073/pnas.012582499 PMC128573 11875207

[29] Homayounfar M, Muneepeerakul R, Anderies JM, Muneepeerakul CP. Linking resilience and robustness and uncovering their trade-offs in coupled infrastructure systems. Earth System Dynamics. 2018;9(4):1159–1168. 10.5194/esd-9-1159-2018

[30] ERS U. U.S. Strawberry Industry; 2013. Available from: https://usda.library.cornell.edu/concern/publications/8s45q876k?locale=en.

[31] ERS U. U.S. Tomato Statistics; 2010. Available from: https://usda.library.cornell.edu/concern/publications/br86b356q?locale=en.

[32] Rădulescu CZ, Rădulescu M. A decision support tool based on a portfolio selection model for crop planning under risk. Studies in Informatics and Control. 2012;21(4):377–382.

[33] Guan Z, Wu F, Whidden A. Florida Strawberry Production Costs and Trends. UF IFAS Extension publication. 2017;FE 1013. Available at: http://edis.ifas.ufl.edu/pdffiles/FE/FE101300.pdf.

[34] Florida Department of Agriculture and Consumer Services. Florida Agriculture Overview and Statistics. 2015. Available at: https://www.fdacs.gov/Agriculture-Industry/Florida-Agriculture-Overview-and-Statistics.

[35] NASS. 2017 State Agriculture Overview (Florida). 2017. Available at: https://www.nass.usda.gov/Quick_Stats/Ag_Overview/stateOverview.php?state=florida&year=2017.

[36] Okrent A, Alston J. The demand for disaggregated food-away-from-home and food-at-home products in the United States 2012.

[37] VanSickle J, McAvoy E. Production Budget for Tomatoes Grown in Southwest Florida and in the Palmetto-Ruskin Area of Florida UF IFAS Extension publication. 2015;FE 818. Available at: https://ufdc.ufl.edu/IR00003786/00001.

[38] Guan Z, Wu F, Sargent S. Labor Requirements and Costs for Harvesting Tomatoes. UF IFAS Extension publication. 2017;FE 1026. Available at: https://edis.ifas.ufl.edu/fe1026.

[39] Roka FM, Guan Z. Farm labor management trends in Florida, USA–Challenges and opportunities. International Journal of Agricultural Management. 2018;7(1):1–9.

[40] Sobekova K, Thomsen MR, Ahrendsen BL, et al Market trends and consumer demand for fresh berries. Applied Studies in Agribusiness and Commerce. 2013;7:11–14. 10.19041/Apstract/2013/2-3/1

